# 1-Amino-5-(4-methyl­benzo­yl)-4-(4-methyl­phen­yl)pyrimidin-2(1*H*)-one

**DOI:** 10.1107/S1600536811047301

**Published:** 2011-11-16

**Authors:** Sema Öztürk Yíldírím, Nilgün Özpozan, Ray J. Butcher

**Affiliations:** a Inorganic Chemistry Department, Howard University, Washington, DC 20059, USA; b Department of Physics, Faculty of Sciences, Erciyes University, 38039 Kayseri, Turkey; c Department of Chemistry, Faculty of Sciences, Erciyes University, 38039 Kayseri, Turkey

## Abstract

In the title compound, C_19_H_17_N_3_O_2_, the dihedral angles between the pyrimidine ring and the two benzene rings are 34.87 (12) (for the directly-bonded ring) and 69.57 (12)°. An intra­molecular N—H⋯O hydrogen bond occurs. The crystal packing features inter­molecular N–H⋯O hydrogen bonds.

## Related literature

For the structures of similar biologically active pyrimidines, see: Akkurt *et al.* (2003 ▶, 2004 ▶); Sarípínar *et al.* (2002 ▶); Yíldírím *et al.* (2007 ▶); Önal & Altural (2006 ▶); Önal & Yíldírím (2007 ▶); Yíldírím *et al.* (2007 ▶); Öztürk *et al.* (1997 ▶, 1999 ▶). For the pharmacological properties of pyrimidines, see: Burdge (2000 ▶).
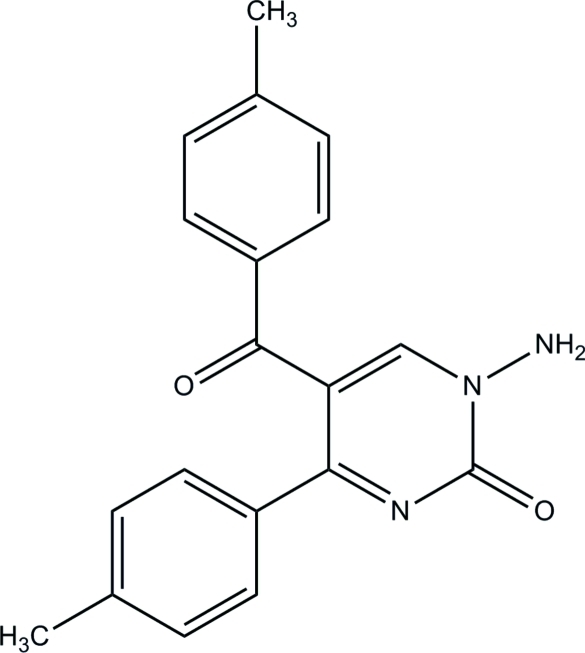


## Experimental

### 

#### Crystal data

C_19_H_17_N_3_O_2_
*M* *_r_* = 319.36
Monoclinic, 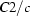

*a* = 24.105 (4) Å
*b* = 5.9547 (10) Å
*c* = 23.170 (4) Å
β = 103.638 (3)°
*V* = 3232.0 (9) Å^3^
*Z* = 8
Mo *K*α radiation
μ = 0.09 mm^−1^
*T* = 150 K
0.20 × 0.07 × 0.06 mm

#### Data collection

Bruker APEXII CCD diffractometer
11340 measured reflections
3296 independent reflections
1803 reflections with *I* > 2σ(*I*)
*R*_int_ = 0.076

#### Refinement

*R*[*F* ^2^ > 2σ(*F* ^2^)] = 0.051
*wR*(*F* ^2^) = 0.136
*S* = 0.96
3296 reflections
227 parameters
H atoms treated by a mixture of independent and constrained refinement
Δρ_max_ = 0.19 e Å^−3^
Δρ_min_ = −0.24 e Å^−3^

### 

Data collection: *APEX2* (Bruker, 2007 ▶); cell refinement: *SAINT* (Bruker, 2007 ▶); data reduction: *SAINT*; program(s) used to solve structure: *SHELXS97* (Sheldrick, 2008 ▶); program(s) used to refine structure: *SHELXL97* (Sheldrick, 2008 ▶); molecular graphics: *ORTEP-3 for Windows* (Farrugia, 1997 ▶); software used to prepare material for publication: *WinGX* (Farrugia, 1999 ▶) and *PLATON* (Spek, 2009 ▶).

## Supplementary Material

Crystal structure: contains datablock(s) global, I. DOI: 10.1107/S1600536811047301/bt5701sup1.cif

Structure factors: contains datablock(s) I. DOI: 10.1107/S1600536811047301/bt5701Isup2.hkl

Supplementary material file. DOI: 10.1107/S1600536811047301/bt5701Isup3.cml

Additional supplementary materials:  crystallographic information; 3D view; checkCIF report

## Figures and Tables

**Table 1:** Hydrogen-bond geometry (Å, °)

*D*—H⋯*A*	*D*—H	H⋯*A*	*D*⋯*A*	*D*—H⋯*A*
N3—H3*A*⋯O2^i^	0.92 (4)	2.20 (3)	3.041 (3)	152 (3)
N3—H3*B*⋯O1	0.92 (3)	2.18 (3)	2.704 (3)	116 (2)
N3—H3*B*⋯O1^ii^	0.92 (3)	2.21 (3)	2.924 (3)	134 (2)

Symmetry codes: (i) 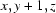; (ii) 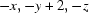.

**Fig. 1.**
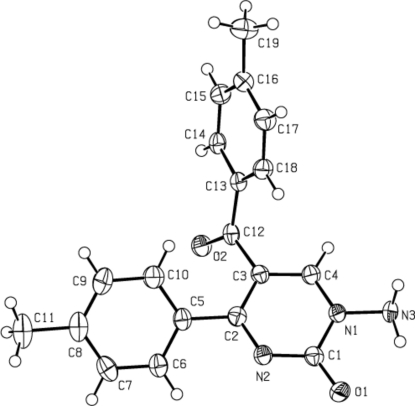
A view of (I) with the atom numbering scheme. Displacement ellipsoids for non-H atoms are drawn at the 50% probability level.

## supplementary crystallographic information

### Comment

As part of our X-ray crystal structure analysis of some compounds of biological interest for a better understanding of the effect of structural and conformational change on biological activity, the structure determination of the title compound was undertaken (Akkurt *et al.*, 2003, 2004; Öztürk *et al.*, 1997, 1999; Yíldírím *et al.*, 2007). 4-Aroyl-5-aryl-2,3-dihydro-2,3-furandiones are obtained starting from 1,3-dicarbonyl compounds with oxalyl halides. In general, 2,3-furandiones are considered convenient and versatile synthons in heterocyclic synthesis. The reactions of the substituted 2,3-furandiones with several semicarbazones, ureas and their thioanalogues and oximes, amides, anilides and hydrazines in different solvents and at various temperatures have been studied recently (Sarípínar *et al.*, 2002). Pyrimidines in general have been of much interest for biological and medical reasons, and thus their chemistry has been investigated extensively (Önal & Altural, 2006; Önal & Yíldírím, 2007). Some are frequently encountered in many drugs used for the treatment of hypothyroidism and hypertension, in cancer chemotherapy or HIV infections (Burdge, 2000).

The title compound has a non planar conformation (Fig. 1). All bond lengths and angles  are in good agreement with those observed in similar compounds (Öztürk *et al.*, 1997, 1999; Yíldírím *et al.*, 2007). The C—N distances have values in the range 1.322 (3) Å -1.408 (3) Å, shorter than the single-bond length of 1.480 Å and longer than the typical C = N distance of 1.280 Å, indicating partial double-bond character and suggesting conjugation in the heterocycle. In spite of this conjugation the pyrimidine ring is slightly distorted from planarity with a maximum deviation of -0.036 (2) Å for atom C1. The mean planes of the rings A (N1/N2/C1–C4), B (C5–C10) and C (C13–C18) make the following dihedral angles with each other: A/B = 34.87 (12), A/C = 69.57 (12) and B/C = 68.74 (12)°. Intermolecular hydrogen-bonding interactions influence the molecular geometry and crystal structure.

### Experimental

In the FT IR spectrum of 1-amino-5-(4-methylbenzoyl)-4-(4-methylphenyl)-1*H*-pyrimidin-2-one, the –NH_2_ absorption band was found to be at 3262 cm^-1^. The C=O absorption band was observed at 1653 cm^-1^. In the ^1^H NMR spectrum of 1-amino-5-(4-methylbenzoyl)-4-(4-methylphenyl)-1*H*-pyrimidin-2-one has a singlet signal at 7.26 p.p.m. assignable to the NH band on the pyrimidine molecule. Finally, the elemental analysis data along with spectroscopic data confirm the structure of 1-amino-5-(4-methylbenzoyl)-4-(4-methylphenyl)-1*H*-pyrimidin-2-one.

20 ml of water and 5 ml of acetic acid were added to a solution of 1 g 5-(4-methylbenzoyl)-1-(methyl-4-methylphenylmethylenamino)-4-(4-methylphenyl) -1*H*-pyrimidin-2-one in 20 ml of ethanol and the mixture was the heated under reflux for 45–50 minutes. With cooling 0.43 g (57%) of 1-amino-5-(4-methylbenzoyl)-4-(4-methylphenyl)-1*H*-pyrimidin-2-one precipitated and was recrystallized from ethanol; m.p.: 471 K; IR (*KBr*): υ= 3250 (–NH_2_), 3036 (aromatic C—H), 2911 (aliphatic C—H), 1680 s (C=O), 1650 s (C=O), 1507–1461 cm^-1^ (C=C and C=N); ^1^H NMR (*DMSO*): δ = 7.71–6.99 (m, 9H, ArH), 7.26 (s, 2H, N—NH_2_), 2.38 p.p.m. (s, 6H, 2xCH_3_). Anal. Calcd. for C_19_H_17_N_3_O_2_: C, 71.45; H, 5.36; N, 13.15. Found: C, 71.19; H, 5.20; N, 12.95.

### Refinement

H atoms bonded to N were freely refined. H atoms bonded to C were refined with C—H = 0.93 and 0.96 Å for aromatic and methyl H, respectively, and constrained to ride on their parent atoms, with *U*_iso_(H) = 1.2 or 1.5*U*_eq_(CH_3_).

### Crystal data

**Table:**

C_19_H_17_N_3_O_2_	*F*(000) = 1344
*M**_r_* = 319.36	*D*_x_ = 1.313 Mg m^−^^3^
Monoclinic, *C*2/*c*	Mo *K*α radiation, λ = 0.71073 Å
Hall symbol: -C 2yc	Cell parameters from 924 reflections
*a* = 24.105 (4) Å	θ = 2.2–20.3°
*b* = 5.9547 (10) Å	µ = 0.09 mm^−^^1^
*c* = 23.170 (4) Å	*T* = 150 K
β = 103.638 (3)°	Needle, colourless
*V* = 3232.0 (9) Å^3^	0.20 × 0.07 × 0.06 mm
*Z* = 8	

### Data collection

**Table:**

Bruker APEXII CCD diffractometer	1803 reflections with *I* > 2σ(*I*)
Radiation source: fine-focus sealed tube	*R*_int_ = 0.076
graphite	θ_max_ = 26.4°, θ_min_ = 1.7°
φ and ω scans	*h* = −30→29
11340 measured reflections	*k* = −7→7
3296 independent reflections	*l* = −28→28

### Refinement

**Table:**

Refinement on *F*^2^	Primary atom site location: structure-invariant direct methods
Least-squares matrix: full	Secondary atom site location: difference Fourier map
*R*[*F*^2^ > 2σ(*F*^2^)] = 0.051	Hydrogen site location: inferred from neighbouring sites
*wR*(*F*^2^) = 0.136	H atoms treated by a mixture of independent and constrained refinement
*S* = 0.96	*w* = 1/[σ^2^(*F*_o_^2^) + (0.0602*P*)^2^] where *P* = (*F*_o_^2^ + 2*F*_c_^2^)/3
3296 reflections	(Δ/σ)_max_ < 0.001
227 parameters	Δρ_max_ = 0.19 e Å^−^^3^
0 restraints	Δρ_min_ = −0.24 e Å^−^^3^

### Special details

**Table:**

Geometry. Bond distances, angles *etc*. have been calculated using the rounded fractional coordinates. All su's are estimated from the variances of the (full) variance-covariance matrix. The cell e.s.d.'s are taken into account in the estimation of distances, angles and torsion angles
Refinement. Refinement of *F*^2^ against ALL reflections. The weighted *R*-factor *wR* and goodness of fit *S* are based on *F*^2^, conventional *R*-factors *R* are based on *F*, with *F* set to zero for negative *F*^2^. The threshold expression of *F*^2^ > σ(*F*^2^) is used only for calculating *R*-factors(gt) *etc*. and is not relevant to the choice of reflections for refinement. *R*-factors based on *F*^2^ are statistically about twice as large as those based on *F*, and *R*- factors based on ALL data will be even larger.

### Fractional atomic coordinates and isotropic or equivalent isotropic displacement parameters (Å^2^)

**Table:**

	*x*	*y*	*z*	*U*_iso_*/*U*_eq_	
O1	0.03523 (7)	0.7379 (3)	0.00058 (7)	0.0342 (6)	
O2	0.05364 (7)	0.0667 (3)	0.20377 (7)	0.0329 (6)	
N1	0.02184 (8)	0.7052 (3)	0.09425 (8)	0.0252 (7)	
N2	0.08437 (8)	0.4603 (3)	0.05992 (9)	0.0284 (7)	
N3	−0.01549 (9)	0.8931 (4)	0.08508 (11)	0.0309 (7)	
C1	0.04687 (10)	0.6364 (4)	0.04807 (11)	0.0269 (8)	
C2	0.09296 (10)	0.3510 (4)	0.11096 (10)	0.0261 (8)	
C3	0.06426 (10)	0.4078 (4)	0.15619 (10)	0.0249 (8)	
C4	0.02914 (10)	0.5911 (4)	0.14525 (11)	0.0267 (8)	
C5	0.13585 (10)	0.1684 (4)	0.11887 (11)	0.0284 (8)	
C6	0.14084 (11)	0.0406 (4)	0.06986 (12)	0.0327 (9)	
C7	0.18031 (11)	−0.1316 (4)	0.07650 (13)	0.0380 (9)	
C8	0.21657 (11)	−0.1806 (4)	0.13099 (13)	0.0396 (10)	
C9	0.21284 (11)	−0.0487 (5)	0.17919 (14)	0.0424 (10)	
C10	0.17293 (11)	0.1214 (5)	0.17364 (12)	0.0368 (9)	
C11	0.25874 (12)	−0.3723 (5)	0.13736 (15)	0.0539 (13)	
C12	0.06651 (10)	0.2659 (4)	0.20998 (11)	0.0265 (8)	
C13	0.08538 (10)	0.3690 (4)	0.26932 (10)	0.0244 (8)	
C14	0.07993 (10)	0.2486 (4)	0.31977 (11)	0.0270 (8)	
C15	0.10127 (10)	0.3342 (4)	0.37568 (11)	0.0320 (8)	
C16	0.13005 (11)	0.5397 (4)	0.38408 (11)	0.0318 (9)	
C17	0.13446 (11)	0.6601 (4)	0.33411 (11)	0.0338 (9)	
C18	0.11219 (11)	0.5777 (4)	0.27750 (11)	0.0310 (8)	
C19	0.15660 (13)	0.6266 (5)	0.44509 (11)	0.0462 (10)	
H3A	0.0032 (13)	0.992 (6)	0.1138 (15)	0.069 (11)*	
H3B	−0.0150 (12)	0.941 (5)	0.0475 (14)	0.062 (10)*	
H4	0.01002	0.63704	0.17370	0.0320*	
H6	0.11755	0.07141	0.03262	0.0392*	
H7	0.18261	−0.21682	0.04352	0.0456*	
H9	0.23761	−0.07503	0.21589	0.0509*	
H10	0.17072	0.20573	0.20681	0.0442*	
H11A	0.27484	−0.37669	0.10323	0.0809*	
H11B	0.28864	−0.35041	0.17247	0.0809*	
H11C	0.23951	−0.51139	0.14043	0.0809*	
H14	0.06172	0.10984	0.31533	0.0323*	
H15	0.09648	0.25381	0.40857	0.0384*	
H17	0.15272	0.79877	0.33872	0.0405*	
H18	0.11516	0.66250	0.24465	0.0372*	
H19A	0.15810	0.78766	0.44415	0.0693*	
H19B	0.13411	0.58008	0.47208	0.0693*	
H19C	0.19458	0.56779	0.45810	0.0693*	

### Atomic displacement parameters (Å^2^)

**Table:**

	*U*^11^	*U*^22^	*U*^33^	*U*^12^	*U*^13^	*U*^23^
O1	0.0454 (11)	0.0280 (10)	0.0284 (10)	0.0028 (9)	0.0072 (8)	0.0049 (8)
O2	0.0430 (11)	0.0207 (10)	0.0339 (10)	−0.0022 (8)	0.0071 (9)	−0.0002 (8)
N1	0.0298 (11)	0.0196 (11)	0.0256 (12)	0.0011 (9)	0.0051 (9)	−0.0007 (9)
N2	0.0340 (12)	0.0214 (11)	0.0291 (12)	0.0003 (10)	0.0061 (10)	0.0002 (9)
N3	0.0376 (13)	0.0215 (12)	0.0330 (13)	0.0094 (10)	0.0069 (11)	0.0045 (11)
C1	0.0306 (14)	0.0208 (13)	0.0291 (14)	−0.0036 (11)	0.0069 (11)	−0.0018 (11)
C2	0.0254 (13)	0.0224 (13)	0.0294 (14)	−0.0029 (11)	0.0042 (11)	−0.0013 (11)
C3	0.0291 (13)	0.0196 (12)	0.0258 (14)	−0.0019 (11)	0.0059 (11)	−0.0015 (10)
C4	0.0306 (14)	0.0208 (13)	0.0284 (14)	−0.0018 (11)	0.0064 (11)	0.0002 (11)
C5	0.0278 (13)	0.0221 (13)	0.0368 (15)	−0.0008 (11)	0.0105 (12)	0.0028 (12)
C6	0.0332 (15)	0.0253 (14)	0.0408 (16)	0.0021 (12)	0.0111 (13)	0.0018 (12)
C7	0.0404 (16)	0.0258 (14)	0.0513 (18)	−0.0018 (13)	0.0179 (14)	−0.0067 (13)
C8	0.0282 (15)	0.0277 (15)	0.064 (2)	0.0020 (12)	0.0131 (14)	0.0063 (14)
C9	0.0325 (16)	0.0420 (18)	0.0507 (19)	0.0086 (14)	0.0057 (14)	0.0090 (15)
C10	0.0319 (15)	0.0405 (17)	0.0379 (16)	0.0048 (13)	0.0079 (12)	−0.0002 (13)
C11	0.0402 (17)	0.0351 (18)	0.088 (3)	0.0102 (14)	0.0186 (17)	0.0089 (17)
C12	0.0261 (13)	0.0187 (13)	0.0346 (15)	0.0012 (11)	0.0072 (11)	0.0007 (11)
C13	0.0254 (13)	0.0204 (13)	0.0275 (13)	0.0017 (10)	0.0067 (10)	0.0005 (11)
C14	0.0274 (13)	0.0203 (13)	0.0345 (15)	0.0019 (11)	0.0099 (11)	0.0042 (11)
C15	0.0369 (15)	0.0317 (15)	0.0297 (14)	0.0023 (13)	0.0125 (12)	0.0046 (12)
C16	0.0328 (15)	0.0323 (15)	0.0310 (15)	0.0022 (12)	0.0087 (12)	−0.0013 (12)
C17	0.0402 (16)	0.0254 (14)	0.0355 (15)	−0.0062 (12)	0.0084 (13)	−0.0026 (12)
C18	0.0417 (15)	0.0212 (13)	0.0300 (14)	−0.0024 (12)	0.0082 (12)	0.0035 (12)
C19	0.0557 (19)	0.0499 (19)	0.0331 (16)	−0.0081 (16)	0.0106 (14)	−0.0065 (15)

### Geometric parameters (Å, °)

**Table:**

O1—C1	1.229 (3)	C13—C14	1.404 (3)
O2—C12	1.226 (3)	C14—C15	1.374 (3)
N1—N3	1.420 (3)	C15—C16	1.398 (3)
N1—C1	1.408 (3)	C16—C17	1.387 (4)
N1—C4	1.338 (3)	C16—C19	1.500 (4)
N2—C1	1.369 (3)	C17—C18	1.385 (4)
N2—C2	1.323 (3)	C4—H4	0.9300
N3—H3A	0.92 (4)	C6—H6	0.9300
N3—H3B	0.92 (3)	C7—H7	0.9300
C2—C3	1.426 (3)	C9—H9	0.9300
C2—C5	1.482 (3)	C10—H10	0.9300
C3—C12	1.496 (3)	C11—H11A	0.9600
C3—C4	1.368 (3)	C11—H11B	0.9600
C5—C10	1.397 (4)	C11—H11C	0.9600
C5—C6	1.396 (4)	C14—H14	0.9300
C6—C7	1.383 (4)	C15—H15	0.9300
C7—C8	1.387 (4)	C17—H17	0.9300
C8—C9	1.386 (4)	C18—H18	0.9300
C8—C11	1.512 (4)	C19—H19A	0.9600
C9—C10	1.382 (4)	C19—H19B	0.9600
C12—C13	1.476 (3)	C19—H19C	0.9600
C13—C18	1.393 (3)		
			
N3—N1—C1	119.06 (19)	C15—C16—C19	121.4 (2)
N3—N1—C4	118.6 (2)	C15—C16—C17	118.0 (2)
C1—N1—C4	122.2 (2)	C17—C16—C19	120.6 (2)
C1—N2—C2	120.9 (2)	C16—C17—C18	121.2 (2)
N1—N3—H3B	103.7 (19)	C13—C18—C17	120.6 (2)
H3A—N3—H3B	112 (3)	N1—C4—H4	120.00
N1—N3—H3A	102 (2)	C3—C4—H4	119.00
O1—C1—N1	119.3 (2)	C5—C6—H6	120.00
O1—C1—N2	123.8 (2)	C7—C6—H6	120.00
N1—C1—N2	116.8 (2)	C6—C7—H7	119.00
N2—C2—C5	115.4 (2)	C8—C7—H7	119.00
C3—C2—C5	121.9 (2)	C8—C9—H9	119.00
N2—C2—C3	122.7 (2)	C10—C9—H9	119.00
C2—C3—C12	123.4 (2)	C5—C10—H10	120.00
C2—C3—C4	116.1 (2)	C9—C10—H10	120.00
C4—C3—C12	120.3 (2)	C8—C11—H11A	109.00
N1—C4—C3	121.0 (2)	C8—C11—H11B	109.00
C2—C5—C6	119.4 (2)	C8—C11—H11C	109.00
C2—C5—C10	122.5 (2)	H11A—C11—H11B	109.00
C6—C5—C10	118.1 (2)	H11A—C11—H11C	109.00
C5—C6—C7	120.2 (2)	H11B—C11—H11C	109.00
C6—C7—C8	121.8 (3)	C13—C14—H14	120.00
C7—C8—C9	117.9 (2)	C15—C14—H14	120.00
C7—C8—C11	120.8 (3)	C14—C15—H15	119.00
C9—C8—C11	121.3 (3)	C16—C15—H15	119.00
C8—C9—C10	121.1 (3)	C16—C17—H17	119.00
C5—C10—C9	120.9 (3)	C18—C17—H17	119.00
C3—C12—C13	118.9 (2)	C13—C18—H18	120.00
O2—C12—C13	121.7 (2)	C17—C18—H18	120.00
O2—C12—C3	119.4 (2)	C16—C19—H19A	109.00
C12—C13—C14	119.6 (2)	C16—C19—H19B	109.00
C12—C13—C18	121.9 (2)	C16—C19—H19C	109.00
C14—C13—C18	118.3 (2)	H19A—C19—H19B	109.00
C13—C14—C15	120.5 (2)	H19A—C19—H19C	109.00
C14—C15—C16	121.3 (2)	H19B—C19—H19C	109.00
			
N3—N1—C1—O1	−1.1 (3)	C2—C5—C6—C7	−179.5 (2)
C4—N1—C1—O1	174.6 (2)	C10—C5—C6—C7	2.1 (4)
N3—N1—C1—N2	177.4 (2)	C2—C5—C10—C9	−179.3 (3)
C4—N1—C1—N2	−7.0 (3)	C6—C5—C10—C9	−0.9 (4)
N3—N1—C4—C3	179.5 (2)	C5—C6—C7—C8	−1.2 (4)
C1—N1—C4—C3	3.8 (4)	C6—C7—C8—C9	−1.0 (4)
C1—N2—C2—C3	0.1 (4)	C6—C7—C8—C11	179.0 (3)
C2—N2—C1—O1	−176.7 (2)	C7—C8—C9—C10	2.2 (4)
C2—N2—C1—N1	4.9 (3)	C11—C8—C9—C10	−177.8 (3)
C1—N2—C2—C5	−178.3 (2)	C8—C9—C10—C5	−1.3 (4)
C5—C2—C3—C4	174.9 (2)	O2—C12—C13—C14	−10.5 (4)
C5—C2—C3—C12	−11.4 (4)	O2—C12—C13—C18	165.1 (2)
N2—C2—C3—C12	170.4 (2)	C3—C12—C13—C14	170.8 (2)
N2—C2—C5—C10	143.4 (2)	C3—C12—C13—C18	−13.6 (4)
C3—C2—C5—C6	146.7 (2)	C12—C13—C14—C15	174.9 (2)
N2—C2—C5—C6	−35.0 (3)	C18—C13—C14—C15	−0.8 (4)
N2—C2—C3—C4	−3.4 (4)	C12—C13—C18—C17	−173.6 (2)
C3—C2—C5—C10	−35.0 (4)	C14—C13—C18—C17	2.1 (4)
C12—C3—C4—N1	−172.6 (2)	C13—C14—C15—C16	−1.5 (4)
C2—C3—C12—O2	−53.6 (3)	C14—C15—C16—C17	2.6 (4)
C4—C3—C12—O2	119.9 (3)	C14—C15—C16—C19	−176.1 (3)
C4—C3—C12—C13	−61.4 (3)	C15—C16—C17—C18	−1.4 (4)
C2—C3—C12—C13	125.2 (3)	C19—C16—C17—C18	177.3 (3)
C2—C3—C4—N1	1.3 (3)	C16—C17—C18—C13	−1.0 (4)

### Hydrogen-bond geometry (Å, °)

**Table:**

*D*—H···*A*	*D*—H	H···*A*	*D*···*A*	*D*—H···*A*
N3—H3A···O2^i^	0.92 (4)	2.20 (3)	3.041 (3)	152 (3)
N3—H3B···O1	0.92 (3)	2.18 (3)	2.704 (3)	116 (2)
N3—H3B···O1^ii^	0.92 (3)	2.21 (3)	2.924 (3)	134 (2)
C19—H19B···N2^iii^	0.9600	2.6100	3.544 (4)	166.00

Symmetry codes: (i) *x*, *y*+1, *z*; (ii) −*x*, −*y*+2, −*z*; (iii) *x*, −*y*+1, *z*+1/2.
